# Comparison of outcomes of chronic kidney disease based on etiology: a prospective cohort study from KNOW-CKD

**DOI:** 10.1038/s41598-023-29844-x

**Published:** 2023-03-02

**Authors:** Hyunjin Ryu, Yeji Hong, Eunjeong Kang, Minjung Kang, Jayoun Kim, Hayne Cho Park, Yun Kyu Oh, Ho Jun Chin, Sue K. Park, Ji Yong Jung, Young Youl Hyun, Su Ah Sung, Curie Ahn, Kook-Hwan Oh, KNOW-CKD Study Group, Curie Ahn, Kook-Hwan Oh, Hajeong Lee, Seung Seok Han, Hyunjin Ryu, Eunjeong Kang, Minjung Kang, Youngok Ko, Jeongok So, Aram Lee, Dong Wan Chae, Yong Jin Yi, Hyun Jin Cho, Jung Eun Oh, Kyu Hun Choi, Seung Hyeok Han, Tae-Hyun Yoo, Mi Hyun Yu, Kyu-Beck Lee, Young Youl Hyun, Hyun Jung Kim, Yong-Soo Kim, Sol Ji Kim, Wookyung Chung, Ji Yong Jung, Kwon Eun Jin, Su Ah Sung, Sung Woo Lee, Hyang Ki Min, Soon Bin Kwon, Soo Wan Kim, Seong Kwon Ma, Eun Hui Bae, Chang Seong Kim, Hong Sang Choi, Minah Kim, Tae Ryom Oh, Sang Heon Suh, Su Hyun Song, Se Jeong Lee, Yeong Hoon Kim, Sun Woo Kang, Hoseok Koo, Tae Hee Kim, Yun Mi Kim, Young Eun Oh, Eun Young Seong, Sang Heon Song, Miyeun Han, Hyo Jin Kim, Seunghee Ji, Tae Ik Chang, Ea Wha Kang, Kyoung Sook Park, Aei Kyung Choi, Ja-Ryong Koo, Jang-Won Seo, Sun Ryoung Choi, Seon Ha Baek, Myung Sun Kim, Yun Kyu Oh, Jeong Mi Park, Byung-Joo Park, Sue K. Park, Joongyub Lee, Choonghyun Ahn, Kyungsik Kim, Jayoun Kim, Dayeon Nam, Soohee Kang, Juhee Lee, Heejung Ahn, Dong Hee Seo, Soyoung Kim, Korea Biobank, Ok Park, Il Yoel Kim, Sung Hyun Kang, Kyoung Hwa Kim

**Affiliations:** 1 Department of Internal Medicine, Seoul National University Hospital, Seoul, Republic of Korea; 2 Rehabilitation Medical Research Center, Korea Workers’ Compensation and Welfare Service Incheon Hospital, Incheon, Republic of Korea; 3 Department of Internal Medicine, Ewha Womans University Medical Center, Seoul, Republic of Korea; 4 Medical Research Collaborating Center, Seoul National University Hospital, Seoul, Republic of Korea; 5 Department of Internal Medicine, Hallym University Kangnam Sacred Heart Hospital, Seoul, Korea; 6 Department of Internal Medicine, Seoul National University Boramae Medical Center, Seoul, Korea; 7 Department of Internal Medicine, Seoul National University College of Medicine, Seoul, 03080 Republic of Korea; 8 Department of Internal Medicine, Seoul National University Bundang Hospital, Seongnam, Republic of Korea; 9 Department of Preventive Medicine, Seoul National University College of Medicine, Seoul, Korea; 10 Division of Nephrology, Department of Internal Medicine, Gachon University of Gil Medical Center, Gachon University School of Medicine, Incheon, Korea; 11 Department of Internal Medicine, Kangbuk Samsung Hospital, College of Medicine, Sungkyunkwan University, Seoul, Korea; 12 Department of Internal Medicine, Eulji Medical Center, Eulji University, Seoul, Korea; 13 Yonsei University Severance Hospital, Seoul, Republic of Korea; 14 Seoul St. Mary’s Hospital, The Catholic University of Korea, Seoul, Republic of Korea; 15 Chonnam National University Hospital, Gwangju, Republic of Korea; 16 Inje University Busan Paik Hospital, Busan, Republic of Korea; 17 Pusan National University Hospital, Busan, Republic of Korea; 18 National Health Insurance Service Ilsan Hospital, Goyang-si, Republic of Korea; 19 Hallym University Dongtan Sacred Heart Hospital, Hwaseong-si, Republic of Korea; 20 LabGenomics, Seongnam, Republic of Korea; 21 Korea Centers for Disease Control and Prevention, Osong, Korea

**Keywords:** Outcomes research, Chronic kidney disease

## Abstract

The causes of chronic kidney disease (CKD) affects its outcomes. However, the relative risks for adverse outcomes according to specific causes of CKD is not well established. In a prospective cohort study from KNOW-CKD, a cohort was analyzed using overlap propensity score weighting methods. Patients were grouped into four categories according to the cause of CKD: glomerulonephritis (GN), diabetic nephropathy (DN), hypertensive nephropathy (HTN), or polycystic kidney disease (PKD). From a total of 2070 patients, the hazard ratio of kidney failure, the composite of cardiovascular disease (CVD) and mortality, and the slope of the estimated glomerular filtration rate (eGFR) decline according to the cause of CKD were compared between causative groups in a pairwise manner. There were 565 cases of kidney failure and 259 cases of composite CVD and death over 6.0 years of follow-up. Patients with PKD had a significantly increased risk for kidney failure compared to those with GN [Hazard ratio (HR) 1.82], HTN (HR 2.23), and DN (HR 1.73). For the composite outcome of CVD and death, the DN group had increased risks compared to the GN (HR 2.07), and HTN (HR 1.73) groups but not to the PKD group. The adjusted annual eGFR change for the DN and PKD groups were − 3.07 and − 3.37 mL/min/1.73 m^2^ per year, respectively, and all of these values were significantly different than those of the GN and HTN groups (− 2.16 and − 1.42 mL/min/1.73 m^2^ per year, respectively). In summary, the risk of kidney disease progression was relatively higher in patients with PKD compared to other causes of CKD. However, the composite of CVD and death was relatively higher in patients with DN-related CKD than in those with GN- and HTN-related CKD.

## Introduction

Chronic kidney disease (CKD), which is rapidly increasing in prevalence and incidence, is a heterogeneous set of diseases caused by various risk factors and comorbid conditions^1–5^. Although CKD patients share similar pathophysiologies involved with the kidney disease progression, the course and speed of CKD progression and associated complications differ according to the underlying causes. Therefore in the KDIGO guidelines, the cause of CKD is considered one of the important predictors of the outcome, together with other variables such as the glomerular filtration rate category, the albuminuria category, and other comorbid conditions^5^. However, the relative risk for adverse outcomes according to the specific cause of CKD has not been well studied^6^. Direct comparisons of outcomes according to the specific cause of CKD are important to understand the natural progression of CKD and to characterize possible complications according to the cause of CKD. This is critical for CKD management during the predialysis period, both to slow progression and to improve long-term outcomes. In addition, this knowledge can help determine high-risk groups among the CKD population so that resources can be prioritized and therapies can be more targeted.

A few studies have investigated relative risks for adverse outcomes in the CKD population according to specific causes^7–10^. However, previous studies did not cover all of the major CKD etiologies or stages or address all major outcomes. Due to the limitations, the results cannot be extrapolated to the entire adult CKD population.

In this study, we analyzed the hazard ratio for kidney progression and the composite outcome of cardiovascular disease (CVD) and all-cause mortality according to the cause of CKD in a prospective cohort. To investigate the effects of the causes of CKD on the outcomes, overlap weighted methods were used to adjust for the possible confounding factors. Additionally, we analyzed the annual rates of estimated glomerular filtration (eGFR) decline according to the cause of CKD to determine kidney progression patterns according to CKD etiology.

## Methods

This was a longitudinal study of a prospective cohort of CKD patients in Korea, called KNOW-CKD (KoreaN cohort study for Outcome in patients With Chronic Kidney Disease). KNOW-CKD is a multicenter prospective cohort study that enrolled adult predialysis patients with CKD stages G1 to G5^11^. Patients were classified into four groups according to the specific cause of CKD at enrollment: glomerulonephritis (GN), diabetic nephropathy (DN), hypertensive nephropathy (HTN), and Polycystic kidney disease (PKD). Each group classification was determined based on pathologic diagnosis if a biopsy result was available (27.6% of total patients: 66.5% of GN group, 6.4% of DN group, 7.3% of HTN group and 1.4% of PKD group). Otherwise, group classifications were based on clinical diagnoses. The biopsy-proven GN consisted as following – 40% IgA nephropathy, 7% focal segment glomerular sclerosis, 6% membranous nephropathy, 5% crescentic GN, 2.4% minimal change disease, and 1.5% lupus nephritis. Non-biopsy-proven GN was defined as the clinical history manifesting chronic GN and the presence of albuminuria or glomerular hematuria with or without an underlying systemic disease causing GN. The active GN population taking immunosuppressant at enrollment was excluded to minimize the heterogeneity by treatment. Diagnosis of DN was strictly based on albuminuria in a patient with type 2 diabetes and the presence of diabetic retinopathy. To exclude DN patients who may have combined GN, diabetic patients with glomerular hematuria were not included in the DN group. HTN was diagnosed by a history of hypertension and the absence of a systemic illness associated with kidney damage. Only the patients with proteinuria < 1.5 g/day and a proportion of urine albumin < 50% of urine protein were included in HTN to exclude the GN population. To diagnose PKD, unified ultrasound criteria were used^12^. Other causative diseases was categorized as ‘unclassified’ and excluded from our analysis.

**Figure 1:**
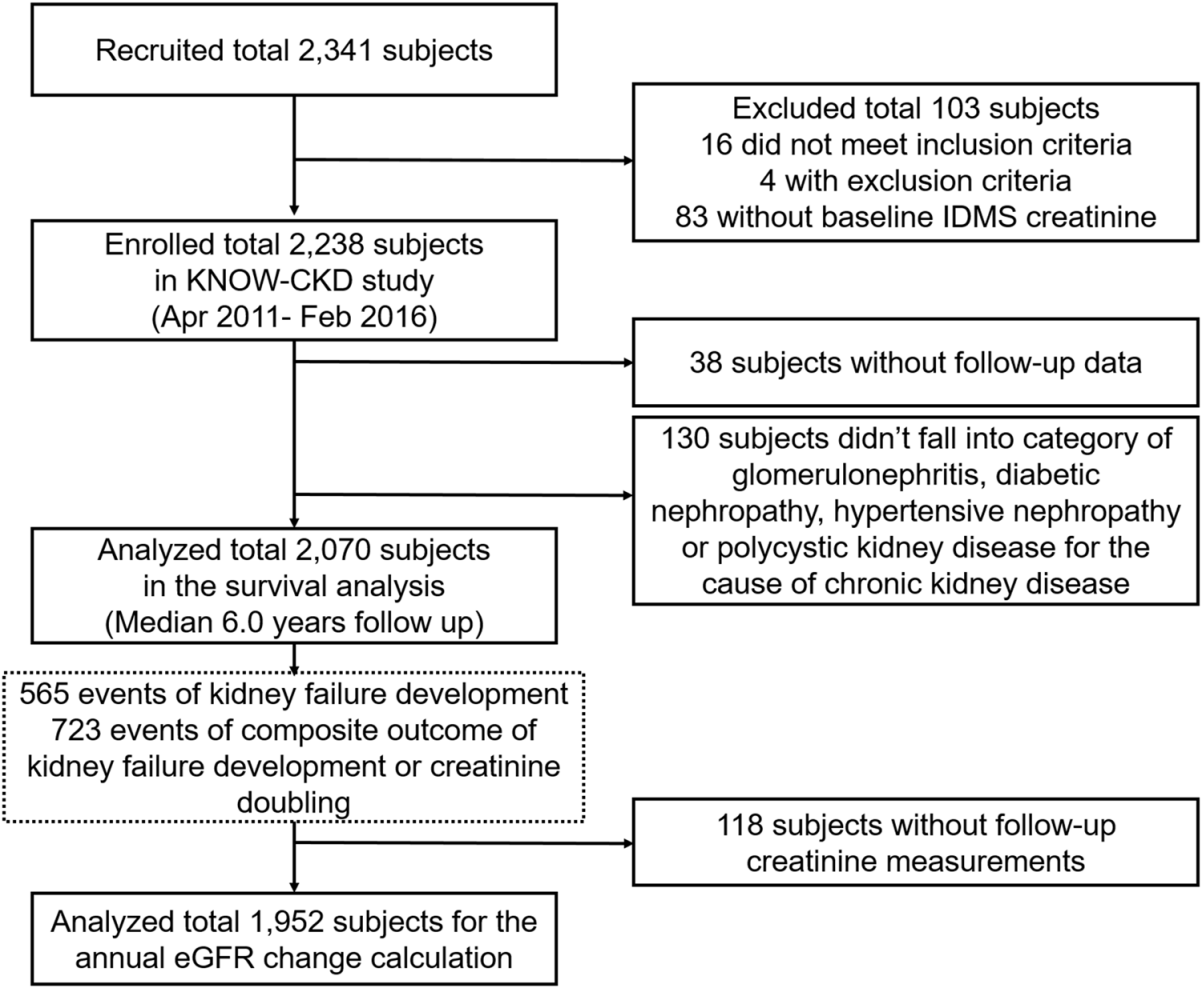
Flowchart of enrolled study patients. eGFR, estimated glomerular filtration rate; IDMS, isotope dilution mass spectrometry.

A total of 2238 patients enrolled in the study from April 2011 to February 2016. After excluding patients with unclassified etiology or without follow-up data, 2070 patients were finally analyzed in this study for survival analysis with follow up until March 31, 2020. To determine the annual eGFR change and trajectory, we included only those patients (n = 1952) with more than two creatinine measurements (Fig. 1). Written informed consent from each patient was collected voluntarily at the time of enrollment. The study was approved by the institutional review board of each participating hospital: Chonnam National University Hospital (CNUH-2011-092), Eulji General Hospital (201105-01), Gil Hospital (GIRBA2553), Kangbuk Samsung Medical Center (2011-01-076), Pusan Paik Hospital (11-091), Seoul National University Bundang Hospital (B-1106/129-008), Seoul National University Hospital (H-1704-025-842), Seoul St. Mary’s Hospital (KC11OIMI0441), and Yonsei University Severance Hospital (4-2011-0163). This study follows the guidelines of the 2008 Declaration of Helsinki.

Demographic details and medication history were collected at enrollment. Serum creatinine was measured at each study visit by a central laboratory (Lab Genomics, Seoul, Republic of Korea) using an isotope dilution mass spectrometry-traceable method. For eGFR, the CKD -EPI equation based on serum creatinine was used^13^. After the baseline visit, patients were followed-up at 6 and 12 months and then every 1 year until death or drop-out and follow-up events were recorded. In case of loss to follow-up, patients were censored for kidney and CVD events at the last follow-up visit. Death and the cause of death were collected using either hospital medical records or data from the National Database of Statistics Korea using the Korean resident registration number. Data were collected until whichever came first: drop-out, death, or March 31, 2020.

Both kidney failure and the composite of kidney failure and/or creatinine doubling were used as kidney outcomes. Kidney failure was defined as starting maintenance dialysis (required for longer than 3 months) or receiving kidney transplantation. Another outcome was the composite outcome of CVD and all-cause death. CVD was defined as any first event of the following that needed hospitalization, intervention, or therapy during the follow-up period : acute myocardial infarction, unstable angina which needed admission due to aggravated coronary ischemic symptoms, percutaneous coronary artery intervention or coronary bypass graft surgery, ischemic or hemorrhagic cerebral stroke, cerebral artery aneurysm, congestive heart failure, symptomatic arrhythmia, aggravated valvular heart meant by requiring hospital admission, any pericardial disease that required hospital admissions such as pericarditis, pericardial effusion, or cardiac tamponade, abdominal aortic aneurysm, or severe peripheral arterial disease (Table S1).

The chi-square test or Anova was used to compare the baseline characteristics. Non-normally distributed variables such as parathyroid hormone, urine protein/creatinine, and high sensitivity C-reactive protein were compared by Kruskal–Wallis test. The four groups had significant differences in baseline characteristics including age and baseline eGFR; we therefore used the overlap propensity score (PS) weighting method to minimize the effects of confounding factors on outcomes^14^. Overlap weighting is a PS method that tries to mimic important attributes of randomized clinical trials. This method can overcome the potential limitation of adjusting the difference in measured characteristics using classic PS methods of inverse probability of treatment weighting (IPTW). Overlap weighting overcomes these limitations by assigning weights to each patient that are proportional to the probability of that patient belonging to the opposite group^15^. PSs were calculated using a logistic model with the following variables since they showed significant differences among the four groups: age, sex, body mass index, CKD stage, mean blood pressure, CVD, hemoglobin, serum uric acid, calcium, phosphorous, albumin, total cholesterol, high-density lipoprotein, low-density lipoprotein, fasting blood sugar, intact parathyroid hormone, urine protein-to-creatinine ratio, high-sensitivity C-reactive protein, diuretics use, statin use, and angiotensin converting enzyme inhibitor or angiotensin receptor blocker use in this study. The log10 transformed values were used for PS calculation with the non-normally distributed variables such as parathyroid hormone, urine protein-to-creatinine ratio, and high sensitivity C-reactive protein. The patients in the compared group were weighted by the probability of the reference group (1-PS), and the patients in the reference group were weighted by the probability of the compared group (PS). For two groups of CKD causes, we applied the overlap weighting method to each set, resulting in a total of 6 sets. To visually compare distributions of balance, the density plots were created (Figure S1). Additionally, the standardized mean difference (SMD) was calculated to check good balance after the overlap weighting method was applied. This is calculated by the absolute value of the difference in mean among groups divided by the standard deviation. The SMD less than or equal to 0.10 means good balance after weighting^15^. In outcome comparison analysis, a Cox proportional hazard model was used for kidney outcomes, and a cause-specific hazard model was used for the composite of CVD and death. In the competing risk model for the composite of CVD and death, kidney failure was considered a competing risk since many patients who started kidney replacement therapy were no longer followed for further event thereafter. Results are presented as hazard ratios (HRs) and 95% confidence intervals (95% CI). To estimate annual eGFR change, generalized linear mixed models were constructed with random intercepts and slopes with an unstructured model for the correlation structure. The results were expressed as estimates (standard errors). In the adjusted models, the variables used in PS score calculation were further adjusted. Spaghetti plots showing the individual trajectories of eGFR during follow-up were drawn to determine patterns of eGFR decline according to cause of CKD. *P* for the quadratic term was tested using polynomial mixed models with random intercepts and slopes. A *P* value less than 0.05 was considered statistically significant. SAS 9.4 (SAS Institute, Cary, NC, USA) and R version 3.5.3 (Foundation for Statistical Computing, Vienna, Austria) were used.

## Results

The mean age of the population was 53.5 ± 12.2 years and 38.7% of subjects were female from a total of 2070 patients. At study entry, the mean eGFR of all total patients was 53.2 ± 30.8 mL/min/1.73 m^2^. By CKD classification, 38.6% of patients were diagnosed with GN, 24.5% with DN, 19.5% with HTN, and 17.4% with PKD.

Baseline characteristics between the four CKD etiology groups showed significant differences (Table 1). All variables showed significant differences among groups and the standardized mean difference were > 0.1 in all variables. In particular, patients with DN (59.2 ± 9.3 years old) or HTN (59.7 ± 10.8 years old) were older than those with GN (49.7 ± 12 years old) or PKD (47 ± 10.7 years old) (*P* < 0.001). The proportion of males and the prevalence of pre-existing CVD was higher in the DN (69.1% and 26.4%, respectively) and HTN (72.6% and 23.8%, respectively) groups than in the GN (55.3% and 7.9%, respectively) and PKD (51% and 6.9%, respectively) groups (*P* < 0.001 for both). Mean eGFR was lower in DN (36.4 ± 21.8 mL/min/1.73 m^2^) and HTN (42.3 ± 21.9 mL/min/1.73 m^2^) groups than GN (60.1 ± 31.4 mL/min/1.73 m^2^) and PKD (72.7 ± 32.8 mL/min/1.73 m^2^) groups (*P* < 0.001). After overlap weighting for each 6 sets, the statistical differences between groups disappeared in all variables (Table S2). Also, SMD was < 0.001 for all variables in all sets which means a good balance between the overlap-weighted sets.

**Table 1:** Comparison of baseline clinical characteristics according to cause of chronic kidney disease before propensity score matching.

	Glomerulonephritis	Hypertensive nephropathy	Diabetic nephropathy	Polycystic kidney disease	*P* value	*SMD*
Number	799	404	507	360		
Age, years	49.7 ± 12.1	59.7 ± 10.8	59.3 ± 9.3	47 ± 10.7	< 0.001	0.74
Female, %	44.6	27.5	31	49.2	< 0.001	0.28
BMI, kg/m^2^	24.2 ± 3.3	25.1 ± 3.5	25.3 ± 3.3	23.5 ± 3	< 0.001	0.32
Mean blood pressure, mmHg	91.6 ± 10.5	94.3 ± 11.8	95.3 ± 12.9	96.8 ± 10.4	< 0.001	0.25
Prevalence of cardiovascular disease, %	7.9	23.8	26.4	6.9	< 0.001	0.35
eGFR, ml/min/1.73 m^2^	60.1 ± 31.4	42.3 ± 21.9	36.4 ± 21.8	72.7 ± 32.8	< 0.001	0.77
Chronic kidney disease stage					< 0.001	0.79
G1, %	21.8	3.7	4.1	35.0		
G2, %	24.3	13.9	7.7	26.9		
G3a, %	16.1	22.5	14.6	13.3		
G3b, %	18.0	27.2	26.0	11.9		
G4, %	15.5	26.2	35.9	9.2		
G5, %	4.3	6.4	11.6	3.6		
Hemoglobin, g/dL	13.2 ± 1.9	13.3 ± 2	11.7 ± 1.8	13.3 ± 1.7	< 0.001	0.44
Uric acid, mg/dL	7.1 ± 1.9	7.3 ± 1.8	7.4 ± 1.9	6.1 ± 1.7	< 0.001	0.39
Calcium, mg/dL	9.1 ± 0.5	9.2 ± 0.5	8.9 ± 0.6	9.3 ± 0.4	< 0.001	0.34
Phosphorous, mg/dL	3.6 ± 0.6	3.6 ± 0.6	4.0 ± 0.8	3.6 ± 0.6	< 0.001	0.30
Albumin, g/dL	4.1 ± 0.4	4.3 ± 0.3	4.0 ± 0.5	4.4 ± 0.3	< 0.001	0.63
Total cholesterol, mg/dL	178.4 ± 38.5	169.1 ± 35.5	167.4 ± 43.8	179.0 ± 33.6	< 0.001	0.19
HDL-cholesterol, mg/dL	51.5 ± 15.6	46.7 ± 14.0	43.6 ± 14.0	54.4 ± 13.9	< 0.001	0.43
LDL-cholesterol, mg/dL	99.9 ± 32.1	94.1 ± 30.5	91.1 ± 34.0	101.6 ± 26.7	< 0.001	0.2
Fasting blood sugar, mg/dL	102.1 ± 24.7	106.7 ± 25.8	136.4 ± 59.0	96.1 ± 11.0	< 0.001	0.57
Parathyroid hormone, pg/mL¶	47.0 (29.8, 69.0)	55.1 (37.9, 86.0)	66.2 (41.6, 120.3)	47.0 (32.9, 70.3)	< 0.001	0.27
Urine protein/creatinine, g/g Cr¶	0.6 (0.3, 1.5)	0.3 (0.1, 0.7)	1.5 (0.5, 3.8)	0.1 (0.1, 0.2)	< 0.001	0.73
Urine albumin/creatinine, mg/g Cr	457.1 (241.1, 1122.9)	207.9 (28.9, 531.8)	1015.6 (276.3, 2584.1)	39.1 (13.4, 131.0)	< 0.001	0.76
Hs-CRP, mg/dL¶	0.7 (0.3, 1.3)	0.7 (0.4, 1.8)	0.7 (0.3, 1.7)	0.5 (0.1, 1.3)	< 0.001	0.11
Diuretics use, %	24.9	34.9	56.4	9.4	< 0.001	0.59
Statin use, %	51.6	57.4	63.9	26.9	< 0.001	0.41
ACEI or ARB use, %	89.7	82.2	87.0	78.6	< 0.001	0.18
Follow up duration, years¶	6.0 (4.4, 7.2)	5.0 (4.0, 7.0)	6.0 (4.2, 7.2)	6.6 (4.2, 8.0)		

^¶^Presented as Median (quartile 1, quartile 3) due to non-normal distributions. Otherwise, continuous variables are presented as mean ± standard deviation and categorical variables as proportion.

ACEI, angiotensin-converting enzyme inhibitors; ARB, angiotensin-receptor blockers; BMI, body mass index; eGFR, estimated glomerular filtration rate; HDL, high-density lipoprotein; Hs-CRP, high sensitivity C-reactive protein; LDL, low density lipoprotein; SMD; standardized mean difference.

During the median 6.0 years of follow-up, there were a total of 565 (27.3%) kidney failure events and 723 (34.9%) composite kidney events. There were 259 (12.5%) events of the composite of CVD and death. The specific cause of CVD and death were summarized in Table S3 and S4. In the PKD population, the most common cause of the cardiovascular event was cerebral hemorrhage or operation/interventions due to cerebral aneurysm (32.3%), each classified as hemorrhagic stroke or other cardiovascular events in Table S3. Among death events, infection (30%) was the major cause of death, followed by malignancy (25%), liver failure (15%), and other causes (10%). These outcomes are different compared to those in the DN group, where, among those patients who had the composite CVD outcome and mortality, 43% had acute coronary syndrome, coronary revascularization, ischemic stroke, or heart failure; cerebral hemorrhagic or aneurysm events accounted for only 5.7% of total cases.

Patients with PKD had a significantly increased risk for kidney failure compared to those with GN (HR 1.82, 95% CI 1.25–2.65), HTN (HR 2.23, 95% CI 1.47–3.38), and DN (HR 1.73, 95% CI 1.05–2.86) in Cox regression analysis. However, the DN, HTN, and GN groups did not show a significant difference in the risk of kidney failure or composite kidney outcome compared to each other (Table 2). DN group had increased risks for the composite outcome of CVD and death compared to the GN (HR 2.07, 95% CI 1.23–3.46), and HTN (HR 1.73, 95% CI 1.08–2.78) groups in cause-specific regression analysis. Patients with DN were not at increased risk of the composite of CVD and death compared to patients with PKD (*P* = 0.169). Similar results were obtained for DN in multivariate Cox regression analyses. PKD showed increased risk of the composite of CVD and death compared to GN (HR 3.11, 95% CI 1.59–6.05) and HTN (HR 1.94, 95% CI 1.03–3.65) in the Cox regression analysis. In the cause-specific regression analysis, PKD showed a significantly higher risk of the composite outcome of CVD and death compared to GN (HR 2.84, 95% CI 1.36–5.93) however, the significance disappeared in the set with HTN (P = 0.334) (Table 3).

**Table 2:** Relative risk for kidney outcomes according to the CKD causes.

Cause of CKD	Kidney failure		Composite kidney outcome	
	HR (95% CI)	*P* value	HR (95% CI )	*P* value
Glomerulonephritis	Reference		Reference	
Hypertensive nephropathy	0.88 (0.64–1.21)	0.429	0.80 (0.60–1.07)	0.129
Diabetic nephropathy	1.20 (0.92–1.57)	0.176	1.18 (0.93–1.49)	0.182
Polycystic kidney disease	1.82 (1.25–2.65)	0.002	2.00 (1.46–2.75)	< 0.001

Cause of CKD	Kidney failure		Composite kidney outcome	
	HR (95% CI)	*P* value	HR (95% CI)	*P* value
Hypertensive nephropathy	Reference		Reference	
Diabetic nephropathy	1.28 (0.94–1.74)	0.111	1.27 (0.96–1.69)	0.093
Polycystic kidney disease	2.23 (1.47–3.38)	< 0.001	2.50 (1.71–3.66)	< 0.001

Cause of CKD	Kidney failure		Composite kidney outcome	
	HR (95% CI)	*P* value	HR (95% CI)	*P* value
Diabetic nephropathy	Reference		Reference	
Polycystic kidney disease	1.73 (1.05–2.86)	0.032	1.87 (1.17–2.97)	0.008

CI, confidence interval; CKD, chronic kidney disease; HR; hazard ratio.

**Table 3:** Relative risk for composite outcome of CVD and all-cause death according to the CKD causes.

Cause of CKD	Cox regression analysis		Cause-specific regression analysis*	
	HR (95% CI)	*P* value	HR (95% CI)	*P* value
Glomerulonephritis	Reference		Reference	
Hypertensive nephropathy	0.97 (0.59–1.59)	0.901	1.24 (0.71–2.15)	0.447
Diabetic nephropathy	1.67 (1.09–2.56)	0.019	2.07 (1.23–3.46)	0.006
Polycystic kidney disease	3.11 (1.59–6.05)	< 0.001	2.84 (1.36–5.93)	0.006

Cause of CKD	Cox regression analysis		Cause-specific regression analysis*	
	HR (95% CI)	*P* value	HR (95% CI)	*P* value
Hypertensive nephropathy	Reference		Reference	
Diabetic nephropathy	1.82 (1.19–2.79)	0.006	1.73 (1.08–2.78)	0.022
Polycystic kidney disease	1.94 (1.03–3.65)	0.041	1.41 (0.70–2.85)	0.334

	Cox regression analysis		Cause-specific regression analysis*	
	HR (95% CI)	*P* value	HR (95% CI)	*P* value
Diabetic nephropathy	Reference		Reference	
Polycystic kidney disease	1.84 (0.93–3.63)	0.079	1.74 (0.79, 3.84)	0.169

*For the composite outcome of CVD and death, cause-specific regression analysis was conducted as a competing risk analysis using the kidney failure as competing event considering the undetected CVD after starting kidney replacement therapy.

CI, confidence interval; CKD, chronic kidney disease; CVD, cardiovascular disease; HR; hazard ratio.

The annual eGFR change of the GN, HTN, DN and PKD groups were − 2.19, − 1.44, − 3.17, and − 3.45 mL/min/1.73 m^2^ per year, respectively. In the adjusted model, the annual eGFR change were − 2.16, − 1.42, − 3.07, and − 3.37 mL/min/1.73 m^2^ per year for the GN, HTN, DN and PKD groups, respectively. The DN and PKD groups had faster rates of decline than the GN and HTN groups, while the HTN group had a slower rate of annual eGFR decline than the GN group (Table 4).

**Table 4:** Annual changes in eGFR according to the cause of CKD.

	Annual changes of eGFR (mL/min/1.73 m^2^ per year)			
	Unadjusted		Adjusted¶	
	Estimate (SE)	*P* value	Estimate (SE)	*P* value
Cause of CKD		< 0.001^a^		< 0.001^a^
Glomerulonephritis	− 2.19 (0.106)		− 2.16 (0.106)	
Hypertensive nephropathy	− 1.44 (0.151)	< 0.001^b^	− 1.42 (0.152)	< 0.001^b^
Diabetic nephropathy	− 3.17 (0.16)	< 0.001^b^ < 0.001^c^	− 3.07 (0.161)	< 0.001^b^ < 0.001^c^
Polycystic kidney disease	− 3.45 (0.155)	< 0.001^b^ < 0.001^c^ 0.201^d^	− 3.37 (0.156)	< 0.001^b^ < 0.001^c^ 0.176^d^

^¶^Adjusted with age, sex, body mass index, CKD stage, mean blood pressure, cardiovascular disease, hemoglobin, uric acid, calcium, phosphorous, albumin, total cholesterol, high-density lipid cholesterol, low-density lipid cholesterol, fasting blood sugar, intact parathyroid hormone, urine protein-to-creatinine ratio, high sensitivity C-reactive protein, diuretics use, statin use, and ACE inhibitor or ARB use.

^a^*P* value for the interaction term between the cause of CKD and time effect in the mixed model.

^b^*P* value for each estimated of CKD causes compared to the glomerulonephritis as reference.

^c^*P* value for each estimated of CKD causes compared to the hypertensive nephropathy as reference.

^d^*P* value for each estimated of CKD causes compared to the diabetic nephropathy as reference.

ACE, angiotensin converting enzyme; ARB, angiotensin receptor blocker; CKD, chronic kidney disease; eGFR, estimated glomerular filtration rate; SE, Standard error.

**Figure 2:**
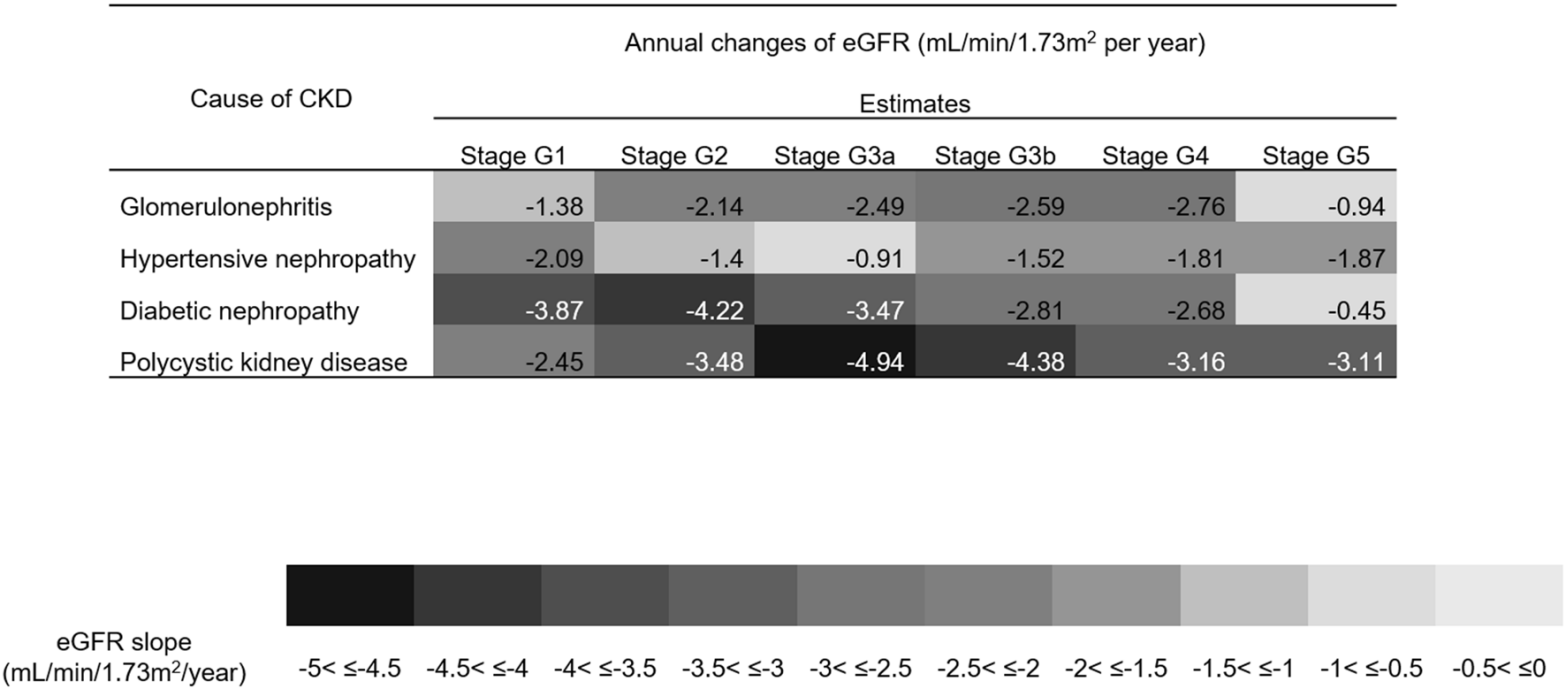
Estimated glomerular filtration rate changes according to baseline CKD stages in each CKD etiology group. CKD of each groups with A1-A3 are classified using eGFR as followed based on KDIGO guideline^30^: stage G1 ≥ 90 mL/min/1.73 m^2^, stage G2 60–89 mL/min/1.73 m^2^, stage G3a 45–59 mL/min/1.73 m^2^, stage G3b 30–44 mL/min/1.73 m^2^, stage G4 15–29 mL/min/1.73 m^2^, and stage G5 < 15 mL/min/1.73 m^2^ without kidney replacement therapy. CKD, chronic kidney disease; eGFR, estimated glomerular filtration rate.

**Figure 3:**
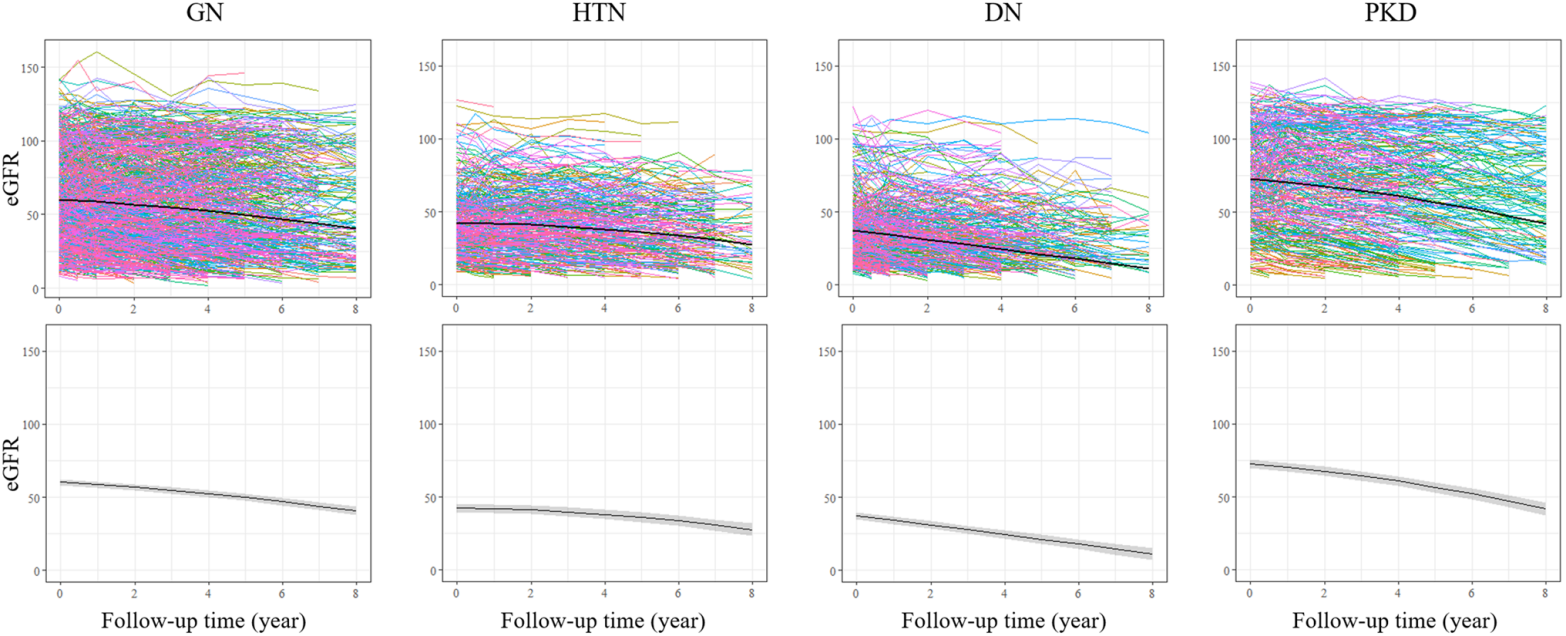
Spaghetti plots (up) and trajectory of eGFR changes (down) during follow-up in each CKD etiology group. Each colors of spaghetti plot indicates trajectory of each patients (up) and the black line represents the mean estimated eGFR trajectory of each groups (with 95% CI represented by the gray shaded area). The trajectory are classified into two types. DN showed a linear decline in eGFR but GN, HTN and PKD showed a convex decline with acceleration of the annual eGFR decline in advanced CKD stages. DN, diabetic nephropathy; eGFR, estimated glomerular filtration rate; GN, glomerulonephritis; HTN, hypertensive nephropathy; PKD, polycystic kidney disease.

The rate of eGFR decline in each group was analyzed according to CKD stage at entry (Fig. 2). The fastest annual decline in eGFR was observed for those patients with PKD stages G3a and G3b (− 4.94 and − 4.38 mL/min/1.73 m^2^ per year, respectively). The overall rate of annual eGFR decline was also fast in the DN group, ranging from − 3.87 to − 2.68 mL/min/1.73 m^2^ per year for stages G1 to G4. In the HTN group, rates of annual eGFR decline were slow for stages G2 to G3b but eGFR declined slightly faster in stages G4 and G5. In the GN group, rates of annual eGFR decline were faster in the more advanced CKD stages. When we visualize the trajectory patterns of eGFR decline, CKD etiologies were classified into two groups: the DN group (*P* for quadratic term = 0.608) group showed a linear decline pattern in eGFR, while the GN, HTN, and PKD groups (*P* for quadratic term < 0.001, for all) showed a convex decline pattern with the acceleration of the annual eGFR decline as the eGFR lowered (Fig. 3).

## Discussion

The KDIGO Guideline states that the cause of CKD should be considered as one of the important predictors of the outcome^5^. A few studies tried to evaluate the outcome difference in the CKD population according to specific causes^7–10^. Post-hoc study of a clinical trial showed that those patients with PKD had a higher risk of kidney failure and a lower risk of death than those with CKD with other etiologies^7^. Studies of the Canadian Study of Prediction of Death, Dialysis and Interim Cardiovascular Events (CanPREDDICT) cohort data reported the relative risks of a few adverse outcomes according to the CKD etiologies^8,9^. The CKD in Children (CKiD) study compared the rate of progression of kidney disease according to the cause of CKD in children^10^. However, the results from these studies are difficult to generalize to the entire CKD population because they include only a subset of the major CKD etiology or analyzed for only some of the major outcomes. This might be due to the difficulty of the study design and analysis technique since many risk factors of kidney disease progression are related to the cause of CKD and can act as potential confounders.

In this prospective cohort study, we compared the relative risks of both kidney outcomes and the composite outcome of CVD and death according to the cause of CKD. Patients were classified into GN, HTN, DN, or PKD groups based either on pathologic diagnoses or clinical judgement criteria at study entry. The baseline characteristics differed according to the cause of CKD in our study population. To overcome this limitation, we used the overlap weighting method. This is particularly advantageous when the comparator groups are initially very different from each other and can achieve good balance and minimize the variances as shown in previous studies^15,16^. Several studies adopted this method to analyze the effect of sex, cancer type, monitoring or treatment on the outcome between groups with significant differences in baseline characteristics^16–20^. By employing this method, we could analyze the hazard ratio for the outcomes between two groups of CKD causes after adjusting for potential confounders. The result showed that patients with PKD had significantly increased risks for kidney outcomes compared to other CKD causes. Surprisingly, the DN group did not show an increased risk of kidney failure compared to other CKD causes. However, the patients in the DN group showed worse outcomes regarding the composite outcome of CVD and mortality compared to the HTN and GN groups. The high risk of CVD and death in the DN group shown in this study was consistent with other well-known studies^21,22^.

We further analyzed the rates of annual eGFR decline to better understand kidney disease progression patterns according to the specific causes of CKD. The rate of GFR decline was faster in the DN and PKD groups compared to the GN and HTN groups. The DN and HTN population had a similar rate of annual eGFR decline to that shown in previous reports; however, the annual rates of decline of eGFR in the PKD group was relatively slower in our study than those reported in previous studies^23,24^. This could be due to differences in baseline clinical characteristics, including CKD stages, PKD genotypes and/or effects of ethnicity. In this study, there are both early and advanced stage PKD patients in our cohort (about 60% were stage G1 or G2) whereas PKD patients in CRISP and HALT studies only included early stage CKD patients and MDRD study enrolled only advanced stage CKD patients^23,24^.

Although the annual eGFR declining rate in the PKD group was slightly slower than in previous reports, patients in the PKD group showed the poorest kidney outcomes compared to those with other causes of CKD. The annual rate of eGFR decline was the fastest in the PKD population. The PKD group showed an increased risk of kidney failure with HRs of 1.73, 1.8 and 2.2 compared to the DN, GN, and HTN groups, respectively. This is a similar result to previous reports^9,24^. Therefore, more efforts on early detection, assessment, and proper management of PKD-related risk factors such as genotype, kidney volumes, hypertension, and kidney-related complications may improve individual PKD patients’ kidney outcomes in the future.

In our study results, the risk of poor kidney outcome was not increased in the DN group, compared to other CKD causes when the confounding factors were adjusted using the overlap weighting methods. This implies the importance of managing the common risk factors and comorbidities in the DN population. However, the risk of CVD and mortality was significantly increased in the DN group compared to the GN and HTN groups. The high risk of CVD and death in the DN population is well known and there have been efforts to find out effective treatments to improve the outcome. However, the strict control of blood glucose levels showed improvement in kidney outcomes but did not in the CVD or death^21,22^. These differences between CVD and kidney outcomes in DN population shown in the previous studies and in our study suggest that different pathophysiology would exist between CVD or mortality and kidney progression.

The CVD and mortality risk was also higher in the PKD population than the GN and HTN population. The PKD group had a similar risk of CVD and mortality to the DN group, but the specific causes of CVD and mortality were different between PKD and DN groups. In the DN group, about 43% of the composite of CVD and mortality were major adverse cardiovascular events such as acute coronary syndrome, coronary revascularization, ischemic stroke, or heart failure which were known major CVD events from the previous studies^25^. However, in the PKD group events due to cerebral aneurysm and infection were the most frequent cause of CVD and death, respectively which correspondence with previous studies^26^.

In this study, we further analyzed eGFR decline patterns according to the cause of CKD. A linear eGFR trajectory was observed in DN group, and the rate of annual eGFR decline was faster in the earlier CKD stages. GN, HTN, and PKD groups showed faster annual eGFR declining in advanced CKD stages. This result is similar to the previous studies that reported trajectory eGFR decline of DN and PKD^23,27^. In DN population, there was a linear association between eGFR and age over time in overall^27^. In PKD population, a non-linear curved eGFR trajectory was seen regardless of the kidney growth rate^23^. In this study, we observed the kidney deterioration pattern and acceleration time differed according to the cause of CKD using a prospective longitudinal cohort. Therefore, patient follow-up and monitoring strategies should be individualized according to the CKD stages, and cause of CKD.

This study has several advantages over existing studies in providing important information about the natural course of CKD progression and extrarenal complications according to CKD etiology. Here, we provided basic information about the hazard ratio of major outcomes from four major causes of CKD within a prospective cohort followed over a long-term period. Robust and up-to-date statistical methods were used to compare the relative risk of major outcomes by the causes of CKD after adjusting for possible confounders. The group size of PKD and GN were big enough for the comparison and statistics. CKD patients older than 18 years with any CKD stage were enrolled to reflect the overall CKD population.

Although, there are several limitations to this study. Baseline characteristics differed among groups. Therefore we used the overlap weighting method to adjust for possible confounding factors. However, there may still have been residual confounders that affected our findings. Furthermore, the etiologic diagnoses of CKD were based on clinical criteria rather than pathologic diagnoses for many patients. Thus, some clinically diagnosed patients might have been misclassified. The issue due to cross-group events, such as the occurrence of new GNs during follow-up in patients in the HTN group, was not considered in the analysis of this study. We could not analyze the risk of each individual type of cardiovascular event or focused on major adverse cardiovascular events since the overall incidence of CV events was relatively lower in our cohort, compared to the Western CKD cohorts^25,28^. The overall CVD incidence is similar to that of the Japanese CKD cohort (CKD-JAC) and this might be the characteristic of the Asian CKD population^29^. Since this is a result from a cohort of Asian CKD patients, the present study warrants further investigation for patients of non-Caucasian ethnicity. Finally, a group with a different composition of GN subtypes may have shown different hazard ratio compared to the other CKD groups evaluated in this study.

We found that patients with PKD had higher risk of kidney progression than patients with DN, GN, or HTN. After adjustment, the DN group did not show an increased risk of kidney failure but had a higher risk of CVD and mortality than patients with GN and HTN. Our findings support the importance of individualized monitoring and management of CKD patients based on the etiology and stage of CKD.

## Supplementary Information

Supplementary Information.

## Data Availability

The datasets generated during and/or analysed during the current study are available from the corresponding author on reasonable request.
