# Author Correction: Comparison of outcomes of chronic kidney disease based on etiology: a prospective cohort study from KNOW-CKD

**DOI:** 10.1038/s41598-023-31613-9

**Published:** 2023-03-21

**Authors:** Hyunjin Ryu, Yeji Hong, Eunjeong Kang, Minjung Kang, Jayoun Kim, Hayne Cho Park, Yun Kyu Oh, Ho Jun Chin, Sue K. Park, Ji Yong Jung, Young Youl Hyun, Su Ah Sung, Curie Ahn, Kook-Hwan Oh, KNOW-CKD Study Group, Curie Ahn, Kook-Hwan Oh, Hajeong Lee, Seung Seok Han, Hyunjin Ryu, Eunjeong Kang, Minjung Kang, Youngok Ko, Jeongok So, Aram Lee, Dong Wan Chae, Yong Jin Yi, Hyun Jin Cho, Jung Eun Oh, Kyu Hun Choi, Seung Hyeok Han, Tae-Hyun Yoo, Mi Hyun Yu, Kyu-Beck Lee, Young Youl Hyun, Hyun Jung Kim, Yong-Soo Kim, Sol Ji Kim, Wookyung Chung, Ji Yong Jung, Kwon Eun Jin, Su Ah Sung, Sung Woo Lee, Hyang Ki Min, Soon Bin Kwon, Soo Wan Kim, Seong Kwon Ma, Eun Hui Bae, Chang Seong Kim, Hong Sang Choi, Minah Kim, Tae Ryom Oh, Sang Heon Suh, Su Hyun Song, Se Jeong Lee, Yeong Hoon Kim, Sun Woo Kang, Hoseok Koo, Tae Hee Kim, Yun Mi Kim, Young Eun Oh, Eun Young Seong, Sang Heon Song, Miyeun Han, Hyo Jin Kim, Seunghee Ji, Tae Ik Chang, Ea Wha Kang, Kyoung Sook Park, Aei Kyung Choi, Ja-Ryong Koo, Jang-Won Seo, Sun Ryoung Choi, Seon Ha Baek, Myung Sun Kim, Yun Kyu Oh, Jeong Mi Park, Byung-Joo Park, Sue K. Park, Joongyub Lee, Choonghyun Ahn, Kyungsik Kim, Jayoun Kim, Dayeon Nam, Soohee Kang, Juhee Lee, Heejung Ahn, Dong Hee Seo, Soyoung Kim, Korea Biobank, Ok Park, Il Yoel Kim, Sung Hyun Kang, Kyoung Hwa Kim

**Affiliations:** 1 Department of Internal Medicine, Seoul National University Hospital, Seoul, Republic of Korea; 2 Rehabilitation Medical Research Center, Korea Workers’ Compensation and Welfare Service Incheon Hospital, Incheon, Republic of Korea; 3 Department of Internal Medicine, Ewha Womans University Medical Center, Seoul, Republic of Korea; 4 Medical Research Collaborating Center, Seoul National University Hospital, Seoul, Republic of Korea; 5 Department of Internal Medicine, Hallym University Kangnam Sacred Heart Hospital, Seoul, Korea; 6 Department of Internal Medicine, Seoul National University Boramae Medical Center, Seoul, Korea; 7 Department of Internal Medicine, Seoul National University College of Medicine, Seoul, 03080 Republic of Korea; 8 Department of Internal Medicine, Seoul National University Bundang Hospital, Seongnam, Republic of Korea; 9 Department of Preventive Medicine, Seoul National University College of Medicine, Seoul, Korea; 10 Division of Nephrology, Department of Internal Medicine, Gachon University of Gil Medical Center, Gachon University School of Medicine, Incheon, Korea; 11 Department of Internal Medicine, Kangbuk Samsung Hospital, College of Medicine, Sungkyunkwan University, Seoul, Korea; 12 Department of Internal Medicine, Eulji Medical Center, Eulji University, Seoul, Korea; 13 Yonsei University Severance Hospital, Seoul, Republic of Korea; 14 Seoul St. Mary’s Hospital, The Catholic University of Korea, Seoul, Republic of Korea; 15 Chonnam National University Hospital, Gwangju, Republic of Korea; 16 Inje University Busan Paik Hospital, Busan, Republic of Korea; 17 Pusan National University Hospital, Busan, Republic of Korea; 18 National Health Insurance Service Ilsan Hospital, Goyang-si, Republic of Korea; 19 Hallym University Dongtan Sacred Heart Hospital, Hwaseong-si, Republic of Korea; 20 LabGenomics, Seongnam, Republic of Korea; 21 Korea Centers for Disease Control and Prevention, Osong, Korea

Correction to: *Scientific Reports* https://doi.org/10.1038/s41598-023-29844-x, published online 02 March 2023

The original version of this Article contained an error in the Funding section.

“This work was supported by the Research Program funded by the Korea Centers for Disease Control and Prevention (2011E3300300, 2012E3301100, 2013E3301600, 2013E3301601, 2013E3301602, 2016E3300200, 2016E3300201, 2016E3300202, 2019E320100, 2019E320101, 2019E320102, and 2022-11-007) and the Bio & Medical Technology Development Program of the National Research Foundation (NRF) & funded by the Korean government (MSIT) (No. 2017M3A9E4044649).”

now reads:

“This work was supported by the Research Program funded by the Korea Disease Control and Prevention Agency (2011E3300300, 2012E3301100, 2013E3301600, 2013E3301601, 2013E3301602, 2016E3300200, 2016E3300201, 2016E3300202, 2019E320100, 2019E320101, 2019E320102, and 2022-11-007) and the Bio & Medical Technology Development Program of the National Research Foundation (NRF) & funded by the Korean government (MSIT) (No. 2017M3A9E4044649).”

The original Article has been corrected.

