# Supplementary material for: Comparison of outcomes of chronic kidney disease based on etiology: a prospective cohort study from KNOW-CKD
Source: Sci Rep. 2023 Mar 2;13:3570. doi: 10.1038/s41598-023-29844-x (PMC9981888; doi:10.1038/s41598-023-29844-x)
Supplement: Supplementary file 1 — Supplementary Information. [file 41598_2023_29844_MOESM1_ESM.docx]

**Supplementary Material**

**Figure S1. Distributions of propensity scores in each set of two groups of CKD causes. The grey bar indicates the reference CKD cause group.**

Each set compared two groups of CKD causes as follows: set 1 between GN and HTN, set 2 between GN and DN, set 3 between GN and PKD, set 4 between HTN and DN, set 5 between HTN and PKD, and set 6 between DN and PKD

DN, diabetic nephropathy; GN, glomerulonephritis; HTN, hypertensive nephropathy; PKD, polycystic kidney disease

**Table S1. Summary of the definition, considered competing events and censoring event of each outcome**

NA, not applicable

**Table S2. Overlap propensity score–weighted baseline characteristics between two causes of chronic kidney disease**

The log10 transformed parathyroid hormone, urine protein/creatinine, and high sensitivity C-reactive protein were used for the PS modeling since they showed non-normally distribution.

In all sets, SMD <0.001 for all variables except variables marked with ¶.

¶ Presented as Median (interquartile range) due to non-normal distributions. Otherwise, continuous variables are presented as mean ± standard deviation and categorical variables as proportion, %.

BMI, body mass index; eGFR, estimated glomerular filtration rate; HbA1C, hemoglobin A1C; HDL, high density lipid; Hs-CRP, high sensitivity-C reactive protein; IPTW, inverse probability of treatment weighting; LDL, low density lipid; SMD; standardized mean difference

**Table S3. The specific cause of cardiovascular disease and death according to the cause of CKD groups during follow-up**

AMI, acute myocardial infarction; CABG, coronary bypass graft surgery; DN, diabetic nephropathy; GN, glomerulonephritis; HTN, hypertensive nephropathy; PCI, percutaneous coronary artery intervention; PKD, polycystic kidney disease

^¶^Other fatal and non-fatal cardiovascular events include fatal and non-fatal events due to deteriorating valvular heart disease, pericardial disease, abdominal aortic aneurysm and cerebral aneurysm (excluding cerebral hemorrhage) requiring hospitalization, intervention, or therapy during follow-up.

**Table S4. The specific cause of death according to each cause of CKD groups**

DN, diabetic nephropathy; GN, glomerulonephritis; HTN, hypertensive nephropathy; PKD, polycystic kidney disease

**Figure S1. Distributions of propensity scores in each set of two groups of CKD causes. The grey bar indicates the reference CKD cause group.**

**
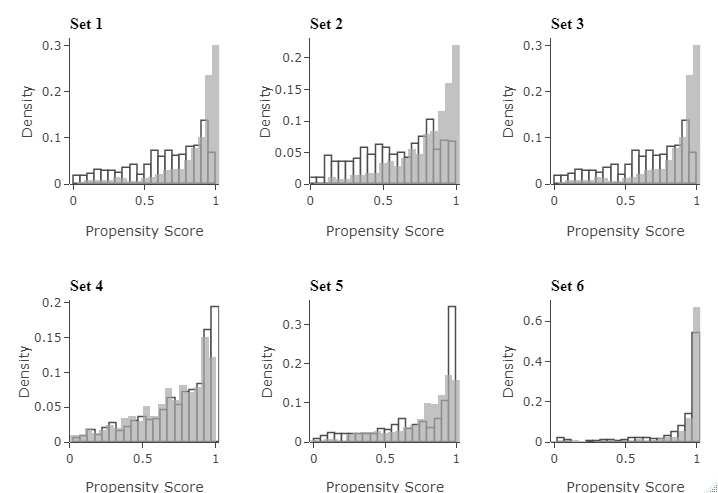
**

Each set compared two groups of CKD causes as follows: set 1 between GN and HTN, set 2 between GN and DN, set 3 between GN and PKD, set 4 between HTN and DN, set 5 between HTN and PKD, and set 6 between DN and PKD

DN, diabetic nephropathy; GN, glomerulonephritis; HTN, hypertensive nephropathy; PKD, polycystic kidney disease

**Table S1. Summary of the definition, considered competing events and censoring event of each outcome**

|  | **Definition** | **Competing event** | **Censoring event** |
| --- | --- | --- | --- |
| **Kidney failure** | Starting maintenance dialysis (required longer than 3 months) or receiving kidney transplantation | NA | Follow-up loss |
| **Composite of kidney failure and/or creatinine doubling** | Kidney failure defined as above and/or creatinine doubling from baseline | NA | Follow-up loss |
| **Composite of cardiovascular disease and mortality** | Any first event of the following that needed hospitalization, intervention, or therapy during the follow-up: acute myocardial infarction, unstable angina which needed admission due to aggravated coronary ischemic symptoms, percutaneous coronary artery intervention or coronary bypass graft surgery, ischemic or hemorrhagic cerebral stroke, cerebral artery aneurysm, congestive heart failure, symptomatic arrhythmia, aggravated valvular heart meant by requiring hospital admission, any pericardial disease that required hospital admission such as pericarditis, pericardial effusion, or cardiac tamponade, abdominal aortic aneurysm, or severe peripheral arterial disease and/or death | Kidney failure | Follow-up loss |

NA, not applicable

**Table S2. Overlap propensity score–weighted baseline characteristics between two causes of chronic kidney disease**

|  | | **Glomerulonephritis** | **Hypertensive nephropathy** | **Glomerulonephritis** | **Diabetic Nephropathy** | **Glomerulonephritis** | **Polycystic kidney disease** | **Hypertensive nephropathy** | **Diabetic Nephropathy** | **Hypertensive nephropathy** | **Polycystic kidney disease** | **Diabetic Nephropathy** | **Polycystic kidney disease** |
| --- | --- | --- | --- | --- | --- | --- | --- | --- | --- | --- | --- | --- | --- |
|  | | **Set 1** | | **Set 2** | | **Set 3** | | **Set 4** | | **Set 5** | | **Set 6** | |
|  | | 163.50 | 163.50 | 152.67 | 152.67 | 127.49 | 127.49 | 124.17 | 124.17 | 92.06 | 92.06 | 41.47 | 41.47 |
| **Age, years** | | 56.3 ± 11.3 | 56.3 ± 10.4 | 57.1 ± 9.7 | 57.1 ± 10.7 | 48.6 ± 10.9 | 48.6 ± 12.1 | 60.5 ± 8.8 | 60.5 ± 10.7 | 54.1 ± 9.1 | 54.1 ±11.3 | 55.3 ± 9.3 | 55.3 ± 9.9 |
| **Female, %** | | 35.6 | 35.6 | 37.4 | 37.4 | 44.9 | 44.9 | 32.8 | 32.8 | 37.5 | 37.5 | 45.6 | 45.6 |
| **BMI, kg/m^2^** | | 24.7 ± 3.1 | 24.7 ± 3.1 | 24.9 ± 3.3 | 24.7 ± 3.6 | 23.7 ± 3.0 | 23.7 ± 3.2 | 25.3 ± 3.1 | 25.3 ± 3.7 | 24.3 ± 2.8 | 24.3 ± 3.8 | 24.1 ± 2.9 | 24.1 ± 2.8 |
| **Mean blood pressure, mmHg** | | 93.1 ± 11.9 | 93.1 ± 10.5 | 93.1 ±11.4 | 93.1 ±10.2 | 94.5 ±10.0 | 94.5 ±11.5 | 94.4 ± 12.5 | 94.4 ± 12.2 | 95.2 ± 10.3 | 95.2 ± 11.9 | 94.6 ± 10.7 | 94.6 ± 13.8 |
| **Prevalence of cardiovascular disease, %** | | 15.4 | 15.4 | 17.3 | 17.3 | 7.5 | 7.5 | 25.6 | 25.6 | 11.4 | 11.4 | 14.3 | 14.3 |
| **eGFR, ml/min/1.73m^2^** | | 44.3 ± 24.6 | 44.3 ± 23.7 | 44.5 ± 26.2 | 44.5 ± 26.8 | 62.5 ± 31.4 | 62.5 ± 32.0 | 38.6 ± 38.6 | 38.6 ± 23.1 | 47.9 ± 24.5 | 47.9 ± 26.3 | 46.7 ± 27.2 | 46.7 ± 27.0 |
| **Chronic kidney disease stage** | |  |  |  |  |  |  |  |  |  |  |  |  |
|  | **G1, %** | 6.5 | 6.5 | 9.0 | 9.0 | 24.3 | 4.8 | 4.8 | 7.3 | 7.3 | 8.8 | 8.8 | 8.8 |
|  | **G2, %** | 15.9 | 15.9 | 14.3 | 14.3 | 25.7 | 9.6 | 9.6 | 19.7 | 19.7 | 18.8 | 18.8 | 18.8 |
|  | **G3a, %** | 19.6 | 19.6 | 15.9 | 15.9 | 14.5 | 15.6 | 15.6 | 22.6 | 22.6 | 16.5 | 16.5 | 16.5 |
|  | **G3b, %** | 25.6 | 25.6 | 24.7 | 24.7 | 16.8 | 26.8 | 26.8 | 22.3 | 22.3 | 21.9 | 21.9 | 21.9 |
|  | **G4, %** | 25.0 | 25.0 | 26.9 | 26.9 | 13.6 | 33.3 | 33.3 | 20.0 | 20.0 | 23.8 | 23.8 | 23.8 |
|  | **G5, %** | 7.4 | 7.4 | 9.1 | 9.1 | 5.1 | 10.0 | 10.0 | 8.0 | 8.0 | 10.1 | 10.1 | 10.1 |
| **Hemoglobin, g/dL** | | 13.1 ±2.1 | 13.1 ± 1.9 | 12.2 ± 1.9 | 12.2 ± 1.8 | 13.1 ± 1.9 | 13.1 ± 1.8 | 12.4 ± 1.9 | 12.4 ± 1.9 | 13.1 ± 1.9 | 13.1 ± 2.0 | 12.2 ± 2.1 | 12.2 ± 1.8 |
| **Uric acid, mg/dL** | | 7.4 ± 1.7 | 7.4 ± 2.0 | 7.2 ± 1.9 | 7.2 ± 1.9 | 6.6 ± 1.8 | 6.6 ± 1.9 | 7.4 ± 1.9 | 7.4 ± 1.8 | 7.1 ± 1.7 | 7.7 ± 1.8 | 6.8 ± 1.6 | 6.8 ± 1.8 |
| **Calcium, mg/dL** | | 9.2 ± 0.5 | 9.2 ± 0.5 | 9.0 ± 0.6 | 9.0 ± 0.5 | 9.2 ± 0.5 | 9.2 ± 0.4 | 9.1 ± 0.6 | 9.1 ± 0.5 | 9.2 ± 0.5 | 9.2 ± 0.5 | 9.1 ± 0.5 | 9.1 ± 0.6 |
| **Phosphorous, mg/dL** | | 3.6 ± 0.7 | 3.6 ± 0.6 | 3.8 ± 0.7 | 3.8 ± 0.7 | 3.6 ± 0.6 | 3.6 ± 0.6 | 3.7 ± 0.7 | 3.7 ± 0.7 | 3.7 ± 0.6 | 3.7 ± 0.7 | 3.8 ± 0.7 | 3.8 ± 0.7 |
| **Albumin, g/dL** | | 4.3 ± 0.4 | 4.3 ± 0.3 | 4.1 ± 0.5 | 4.1 ± 0.4 | 4.3 ± 0.3 | 4.3 ± 0.3 | 4.2 ± 0.4 | 4.2 ± 0.4 | 4.4 ± 0.3 | 4.4 ± 0.3 | 4.3 ± 0.3 | 4.3 ± 0.3 |
| **Total Cholesterol, mg/dL** | | 171.3 ± 37.2 | 171.3 ± 34.1 | 170.8 ± 43.5 | 170.8 ± 36.8 | 174.6 ± 34.9 | 174.6 ± 33.0 | 165.0 ± 38.1 | 165.0 ± 36.7 | 171.5 ± 37.1 | 171.5 ± 34.8 | 164.0 ± 36.3 | 164.0 ± 36.8 |
| **HDL-cholesterol, mg/dL** | | 48.2 ± 15.0 | 48.2 ± 14.2 | 46.4 ± 15.5 | 46.4 ± 12.6 | 51.6 ± 13.9 | 51.6 ± 15.7 | 45.7 ± 15.8 | 45.7 ± 12.5 | 49.7 ± 14.0 | 49.7 ± 15.5 | 48.2 ± 13.3 | 48.2 ± 16.5 |
| **LDL-cholesterol, mg/dL** | | 94.4 ± 31.0 | 94.4 ± 28.4 | 93.4 ±35.0 | 93.4 ± 29.4 | 98.3 ± 26.5 | 98.3 ± 29.7 | 90.2 ± 30.7 | 90.2 ± 29.6 | 94.7 ± 26.3 | 94.7 ± 30.4 | 89.8 ± 24.3 | 89.8 ± 33.8 |
| **Fasting blood sugar, mg/dL** | | 104.5 ± 22.7 | 104.5 ±27.2 | 115.7 ± 38.3 | 115.7 ± 48.8 | 97.5 ± 11.3 | 97.5 ± 17.3 | 114.0 ± 33.8 | 114.0 ± 38.5 | 99.6 ± 12.5 | 99.6 ± 13.2 | 101.3 ± 13.5 | 101.3 ± 28.6 |
| **Parathyroid hormone, pg/mL¶** | | 54.0 (36.8, 83.9) | 54.6 (36.5, 83.3) | 55.0 (35.0, 93.3) | 54.6 (36.5, 88.4) | 51.1 (33.0, 74.7) | 51.1 (33.7, 72.5) | 64.2 (40.9, 109.7) | 62.8 (41.0, 99.5) | 54.6 (37.1, 86.9) | 54.0 (38.0, 84.0) | 54.6 (36.0, 95.3) | 61.8 (34.6, 108.9) |
| **Log (Parathyroid hormone)** | | 4.1 ± 0.7 | 4.1 ± 0.7 | 4.1 ± 0.7 | 4.1 ± 0.7 | 3.9 ± 0.7 | 3.9 ± 0.7 | 4.2 ± 0.7 | 4.2 ± 0.7 | 4.1 ± 0.7 | 4.1 ± 0.7 | 4.1 ± 0.8 | 4.1 ± 0.7 |
| **Urine Protein/creatinine, g/g Cr¶** | | 0.5 (0.2, 1.0) | 0.5 (0.2, 1.0) | 0.8 (0.3, 2.6) | 0.9 (0.5, 2.3) | 0.2 (0.1, 0.4) | 0.2 (0.1, 0.5) | 0.5 (0.2, 1.4) | 0.6 (0.3, 1.2) | 0.2 (0.1, 0.4) | 0.2 (0.1, 0.5) | 0.3 (0.2, 0.6) | 0.3 (0.2, 0.7) |
| **Log (Urine Protein/creatinine)** | | -0.9 ± 1.4 | -0.9 ±1.3 | -0.1 ± 1.4 | -0.1 ±1.3 | -0.5 ± 1.5 | -0.5 ± 1.2 | -0.6 ± 1.3 | -0.6 ± 1.2 | -1.8 ± 1.1 | -1.8 ± 1.5 | -1.2 ± 1.1 | -1.2 ± 1.2 |
| **Hs-CRP, mg/dL¶** | | 0.7 (0.3, 1.7) | 0.7 (0.3, 1.7) | 0.7 (0.3, 1.5) | 0.7 (0.3, 1.3) | 0.6 (0.1, 1.6) | 0.5 (0.2, 1.2) | 0.7 (0.3, 1.7) | 0.7 (0.3, 1.8) | 0.7 (0.3, 1.7) | 0.7 (0.3, 1.4) | 0.6 (0.1, 1.6) | 0.7 (0.2, 1.4) |
| **Log (hs-CRP)** | | -0.3 ± 1.4 | -0.3 ± 1.3 | -0.4 ± 1.2 | -0.4 ± 1.2 | -0.5 ± 1.5 | -0.5 ± 1.2 | -0.3 ± 1.3 | -0.3 ± 1.4 | -0.4 ± 1.5 | -0.4 ± 1.3 | -0.5 ± 1.6 | -0.5 ± 1.2 |
| **Diuretics use, %** | | 32.6 | 32.6 | 41.8 | 41.8 | 15.4 | 15.4 | 44.9 | 44.9 | 20.8 | 20.8 | 26.6 | 26.6 |
| **Statin use, %** | | 54.1 | 54.1 | 60.3 | 60.3 | 33.2 | 33.2 | 60.9 | 60.9 | 45.7 | 45.7 | 49.5 | 49.5 |
| **ACEI or ARB use, %** | | 85.5 | 85.5 | 89.1 | 89.1 | 84.5 | 84.5 | 86.9 | 86.9 | 84.3 | 84.3 | 88.9 | 88.9 |

The log10 transformed parathyroid hormone, urine protein/creatinine, and high sensitivity C-reactive protein were used for the PS modeling since they showed non-normally distribution.

In all sets, SMD <0.001 for all variables except variables marked with¶

¶ Presented as Median (interquartile range) due to non-normal distributions. Otherwise, continuous variables are presented as mean ± standard deviation and categorical variables as proportion, %

BMI, body mass index; eGFR, estimated glomerular filtration rate; HbA1C, hemoglobin A1C; HDL, high density lipid; Hs-CRP, high sensitivity-C reactive protein; IPTW, inverse probability of treatment weighting; LDL, low density lipid; SMD; standardized mean difference

|  | GN | HTN | DN | PKD | Total population |
| --- | --- | --- | --- | --- | --- |
| Fatal and non-fatal cardiovascular events | | | | | |
| AMI | 2 (4.2%) | 4 (7.0%) | 12 (9.8%) | 1 (3.2%) | 19 (7.3%) |
| Unstable angina | 9 (18.8%) | 2 (3.5%) | 9 (7.3%) | 0 | 20 (7.7%) |
| Heart failure | 0 | 5 (8.8%) | 5 (4.1%) | 1 (3.2%) | 11 (4.2%) |
| PCI or CABG | 2 (4.2%) | 8 (14.0%) | 12 (9.8%) | 1 (3.2%) | 23 (8.8%) |
| Ischemic stroke | 1 (2.1%) | 9 (15.8%) | 15 (12.2%) | 1 (3.2%) | 26 (10.0%) |
| Hemorrhagic stroke | 3 (6.3%) | 1 (1.8%) | 7 (5.7%) | 4 (12.9%) | 14 (5.4%) |
| Carotid artery disease | 1 (2.1%) | 0 | 1 (0.8%) | 0 | 2 (0.8%) |
| Peripheral artery disease | 1 (2.1%) | 2 (3.5%) | 1 (0.8%) | 0 | 4 (1.5%) |
| Symptomatic arrhythmia | 2 (4.2%) | 3 (5.3%) | 5 (4.1%) | 1 (3.2%) | 9 (3.5%) |
| Others^¶^ | 10 (20.8%) | 4 (7.0%) | 14 (11.4%) | 9 (29.0%) | 38 (14.7%) |
| Other causes of death | | | | | |
| Infection | 4 (8.3%) | 6 (10.5%) | 12 (9.8%) | 3 (9.7%) | 25 (9.7%) |
| Malignancy | 4 (8.3%) | 5 (8.8%) | 2 (1.6%) | 4 (12.9%) | 15 (5.8%) |
| Sudden cardiac death | 0 | 0 | 2 (1.6%) | 0 | 2 (0.8%) |
| Liver disease | 0 | 3 (5.3%) | 1 (0.8%) | 0 | 4 (1.5%) |
| Others | 2 (4.2%) | 2 (3.5%) | 3 (2.4%) | 0 | 7 (2.7%) |
| Unknown | 7 (14.6%) | 3 (5.3%) | 22 (17.9%) | 6 (19.3%) | 38 (14.7%) |
| Total composite events | 48 | 57 | 123 | 31 | 259 |

**Table S3. The specific cause of cardiovascular disease and death according to the cause of CKD groups during follow-up**

AMI, acute myocardial infarction; CABG, coronary bypass graft surgery; DN, diabetic nephropathy; GN, glomerulonephritis; HTN, hypertensive nephropathy; PCI, percutaneous coronary artery intervention; PKD, polycystic kidney disease

^¶^Other fatal and non-fatal cardiovascular events include fatal and non-fatal events due to deteriorating valvular heart disease, pericardial disease, abdominal aortic aneurysm and cerebral aneurysm (excluding cerebral hemorrhage) requiring hospitalization, intervention, or therapy during follow-up.

**Table S4. The specific cause of death according to each cause of CKD groups**

|  | **Cardiovascular event** | **Infection** | **Malignancy** | **Sudden cardiac death** | **Liver disease** | **Accident** | **Others** | **Unknown** | **All-cause death** |
| --- | --- | --- | --- | --- | --- | --- | --- | --- | --- |
| **GN** | 1 (4.8%) | 5 (23.8%) | 5 (23.8%) | 0 | 0 | 0 | 3 (14.3%) | 7 (33.3%) | 21 |
| **HTN** | 5 (26.3%) | 3 (15.8%) | 4 (21.1%) | 0 | 0 | 1 (5.3%) | 0 | 6 (31.6%) | 19 |
| **DN** | 13 (19.7%) | 16 (24.2%) | 2 (3.03%) | 7 (10.6%) | 1 (1.5%) | 0 | 3 (4.6%) | 24 (36.7%) | 66 |
| **PKD** | 1 (5.0%) | 6 (30.0%) | 5 (25.0%) | 0 | 3 (15.0%) | 0 | 2 (10.0%) | 3 (15.0%) | 20 |
| **Total population** | 20 (15.9%) | 30 (23.8%) | 16 (12.7%) | 7 (5.6%) | 4 (3.2%) | 1 (0.8%) | 8 (6.4%) | 40 (31.8%) | 126 |

DN, diabetic nephropathy; GN, glomerulonephritis; HTN, hypertensive nephropathy; PKD, polycystic kidney disease
